# Personality Traits and Their Association With Falls and Fall‐Related Psychological Concerns in Adults Aged 50 and Older: A Scoping Review

**DOI:** 10.1002/hsr2.72138

**Published:** 2026-03-30

**Authors:** Henrietha C. Adandom, Henry C. Nwankwo, Israel I. Adandom, Olayinka Akinrolie, David R. Scott, Adesola C. Odole, Lisa L. Cook, Gongbing Shan, Oluwagbohunmi A. Awosoga

**Affiliations:** ^1^ Faculty of Health Sciences University of Lethbridge Lethbridge Alberta Canada; ^2^ Centre for Health Economics at Warwick, Clinical Trials Unit University of Warwick, A035, Warwick Medical School Coventry UK; ^3^ Emerging Researchers and Professionals in Ageing ‐ African Network London Ontario Canada; ^4^ Lifespan Brain Health Lab, Department of Psychology University of Calgary Calgary Alberta Canada; ^5^ Library University of Lethbridge Lethbridge Alberta Canada; ^6^ Department of Physiotherapy, College of Medicine University of Ibadan Oyo State Nigeria; ^7^ Health Intelligence, Evidence & Improvement, Primary Care Alberta Lethbridge Alberta Canada; ^8^ Department of Kinesiology & Physical Education University of Lethbridge Lethbridge Alberta Canada

**Keywords:** Accidental Falls, Five‐Factor Model, Personality, Self‐efficacy

## Abstract

**Background and Aims:**

Falls remain a major health challenge in aging, yet little is known about how personality traits influence falls risk and related psychological concerns. This scoping review mapped existing evidence on relationships among personality traits, falls, fear of falling, and fall self‐efficacy in older adults to identify key associations and research gaps.

**Methods:**

A comprehensive search of five databases (MEDLINE, APA PsycINFO, Web of Science, CINAHL, SPORTDiscus) was conducted from inception (1945) through December 2024. Eligible studies examined personality traits, assessed with validated instruments, in relation to falls, fear of falling, or fall self‐efficacy in older adults (≥ 50 years). All empirical designs were included. Data extraction followed PRISMA‐ScR guidance, and findings were synthesized descriptively.

**Results:**

Of 8060 records screened, eight studies met the inclusion criteria (three longitudinal, five cross‐sectional). High neuroticism and low conscientiousness were the most consistently associated with greater fall risk and higher fear of falling. Extraversion showed generally protective associations with fear of falling, while Type A behaviour predicted higher fall incidence among men but not women. Openness and agreeableness showed no consistent patterns. Evidence on fall self‐efficacy was limited to one study, and none addressed balance confidence. Measurement heterogeneity across personality and fall‐related constructs limited comparability across studies.

**Conclusion:**

Personality traits, particularly emotional instability and conscientiousness, appear relevant to fall risk and psychological concerns, though evidence remains sparse. Key gaps include limited work on fall self‐efficacy and balance confidence, under‐representation of clinical populations, and inconsistent measurement approaches. Future studies should use standardized instruments, longitudinal designs, and broader personality frameworks to inform personalised fall prevention strategies.

## Summary

- Neuroticism (emotional instability) and conscientiousness are consistently linked to fall risk and fear of falling in older adults.
- Extraversion showed a generally protective association with fear of falling, though evidence remains limited in scope.
- Standardized personality and fall‐related assessments are needed to guide personality‐informed fall‐prevention interventions.

## 1 Introduction

Falls among older adults pose significant health and economic challenges, contributing to injuries, reduced mobility, and increased healthcare costs [1, 2, 3, 4]. Decades of research have identified a wide range of intrinsic and extrinsic risk factors, including frailty, multimorbidity, and environmental hazards [5, 6, 7, 8]. Recent biomechanical research further demonstrates that factors such as fall direction, impact velocity, and protective responses influence the likelihood and severity of injury [9], underscoring that falls are multidimensional events shaped by interacting biological, psychological, and environmental factors.

Alongside these physical and environmental contributors, psychological concerns play an important role in fall risk and recovery. Fear of falling and low fall self‐efficacy are well‐established predictors of restricted mobility, loss of confidence, and recurrent falls [10, 11, 12, 13]. However, psychological concerns do not fully explain why some older adults develop intense fear or avoidance behaviours without prior falls, while others remain confident and active even after experiencing falls. This variation suggests that deeper, individual‐level characteristics, such as personality traits, may influence how older adults perceive, interpret, and respond to fall‐related events [14].

Falls are increasingly recognized as a multifactorial phenomenon shaped by biological, psychological, social, and biomechanical factors. The Biopsychosocial Model [15] provides a useful framework for understanding these interacting interactions. Within this model, personality traits may shape how individuals perceive, interpret, and respond to fall‐related events. At the same time, personality expression occurs within this broader health context and may both influence and be influenced by age‐related physical changes, functional decline, injury, or recovery processes [16, 17, 18]. For example, higher neuroticism, characterized by emotional instability and anxiety, has been associated with greater fall risk and heightened fear of falling [19, 20]. Conversely, higher conscientiousness, reflecting self‐discipline and cautious behaviour, has been linked to lower fall risk [19, 21]. Other traits, such as extraversion and agreeableness, may influence activity levels, social engagement, and risk‐taking behaviour, indirectly affecting fall risk [22, 23].

Despite these emerging insights, research on personality and falls remains fragmented. Most fall prevention approaches emphasize physical and biomechanical risk factors, with limited attention to personality‐driven variability in fall outcomes. No existing reviews have systematically synthesized evidence on the interplay among personality traits, falls, and fall‐related psychological concerns. This represents an important gap, as understanding these relationships could explain why individuals with similar biomechanical or health profiles respond differently after a fall and could inform more personalized prevention strategies. To address this gap, this present scoping review synthesizes available evidence on the relationships among personality traits, falls, and fall‐related psychological concerns in older adults. Specifically, it asks: (a) What is the current understanding of the relationship among personality traits and falls or fall‐related psychological concerns? and (b) What mediating factors influence this relationship across diverse populations and environments? By mapping the state of knowledge, this review highlights personality as an underexplored determinant of fall risk and adaptation, offering new insights for clinicians, researchers, and policymakers seeking to design multidimensional and tailored fall prevention strategies.

## 2 Methods and Materials

### 2.1 Protocol and Registration

The methodology adhered to Arksey and O'Malley's five‐stage framework and incorporated methodological extensions by the Joanna Briggs Institute (JBI) [24, 25]. Reporting was guided by the Preferred Reporting Items for Systematic Reviews and Meta‐Analyses extension for Scoping Reviews (PRISMA‐ScR) checklist (Appendix 1) [26, 27]. The protocol was registered on the open science framework and has been published [28].

### 2.2 Eligibility Criteria

Studies were eligible if they were published in English from database inception (1945) through December 2024 and examined the relationship between personality traits and falls or fall‐related psychological concerns (fear of falling, fall self‐efficacy, or balance confidence) in older adults, consistent with how this population was defined within the included studies. Across the evidence base, the youngest participants were aged 50 years (e.g., SHARE dataset), so the findings reflect populations aged 50 and older. Both community‐dwelling and hospitalized populations were considered. All empirical study designs were eligible, including cross‐sectional, longitudinal, and mixed methods approaches, provided personality traits were assessed using psychometrically validated and reliable instruments.

Studies were excluded if they: (a) focused exclusively on psychological conditions or disorders unrelated to falls or its psychological concerns (e.g., depression without reference to falls or fear of falling); (b) examined functional outcomes (e.g., activities of daily living [ADL], instrumental activities of daily living [IADL], or homebound status) without assessing falls or fall‐related psychological concerns; (c) presented only conceptual or theoretical papers without empirical data; (d) were conference abstracts without available full‐text; or (e) were published in languages other than English. Although searches were conducted from database inception (1945) through December 2024, the earliest eligible study identified and included was published in 2004, as no earlier publications met the eligibility criteria. Similarly, no eligible mixed‐methods or qualitative‐only studies that examined personality in relation to falls or its psychological concerns were identified during screening.

### 2.3 Information Sources and Search

A health sciences librarian (DS) conducted the search from inception (1945) to December 2024, across five databases, MEDLINE, APA PsycINFO, Web of Science, CINAHL, and SPORTDiscus, selected for their comprehensive coverage of health, psychology, and falls research [29]. Manual reference list checks of included studies were also completed to capture additional records [30].

The search strategy combined controlled vocabulary (e.g., MeSH terms such as “Personality” and “Accidental Falls”) with free‐text keywords (e.g., “Big Five,” “fear of falling,” “fall self‐efficacy,” “concerns about falling”) to maximize retrieval. Additional terms related to frailty and aging were incorporated to enhance sensitivity, which broadened retrieval substantially. However, many records identified through these expanded terms were later excluded at the full‐text stage because they examined related constructs (e.g., depression or anxiety) without assessing personality in relation to falls or its psychological concerns.

The inclusion criteria were deliberately broad to capture any empirical study, quantitative or qualitative, that examined associations between validated personality measures and falls or fall‐related psychological concerns. The relatively small number of eligible studies, therefore, reflects a genuine scarcity of research directly integrating personality and fall outcomes rather than restrictive methodological parameters.

**Figure 1:**
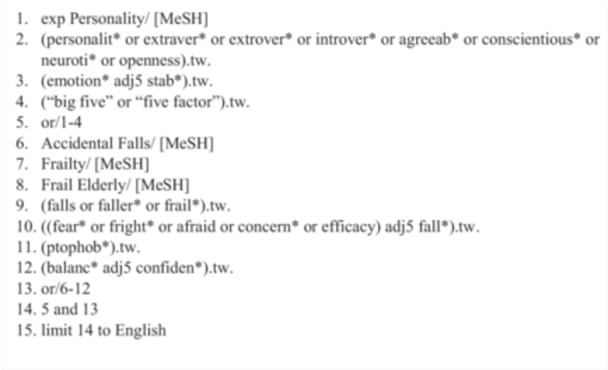
MEDLINE Search Strategy. *Note:* MeSH = medical subject heading; exp = used with a MeSH term to include all narrower MeSH terms; .tw. = field codes for text word; adj# = search for records with terms within # words of each other; quotation marks (e.g., “five factor”) indicate a phrase search; * after keyword indicates truncation (e.g., fall* will retrieve “fall”, “falls”, “falling”, etc.

Search strings were adapted from prior reviews [31, 32, 33, 34] and independently peer‐reviewed using the PRESS checklist [35]. Figure 1 presents the full electronic search strategy for MEDLINE, with detailed strategies for the other databases provided in Appendix 2.

### 2.4 Selection of Source of Evidence

Records were imported into EndNote, where duplicates were removed manually and electronically. Deduplicated records were uploaded into Covidence (https://www.covidence.org) for screening. Two reviewers (IA and HA) independently screened titles and abstracts. Conflicts were resolved through discussion. Full‐text screening was conducted independently by two reviewers (OA and HA), with disagreements adjudicated by a third reviewer (HN). Reasons for exclusion at the full‐text stage (n = 14) are provided as supplementary file (see Appendix 3)

### 2.5 Data Charting Process

The data extraction process was overseen by the lead author (HA), with a second author (IA) independently verifying 100% of extracted data for accuracy. The standardized charting form captured: (a) study details (author, year, country); (b) population characteristics (sample size, demographics); (c) study methodology (design, setting, measures of personality, falls, and their psychological concerns); and (d) key findings (associations between personality and fall‐related outcomes). Discrepancies were resolved by consensus.

### 2.6 Data Items

Primary outcomes were falls and fall‐related psychological concerns, including fear of falling and fall self‐efficacy, informed by prior conceptual work [36]. Table 1 defines all extracted variables.

**Table 1:** Definitions of f key constructs examined in included studies.

Outcomes/variables	Description
Falls	A fall is an event that results in a person coming to rest unintentionally on the ground or other lower level, not as a result of a major intrinsic event such as a stroke, seizure, or loss of consciousness [8].
Fear of falling	Fear of falling is a psychological concern characterized by a sense of anxiety or fear experienced by an individual when faced with the prospect of falling or losing balance while standing, walking, or performing daily activities [37].
Fall self‐efficacy	Refers to an individual's confidence in their ability to avoid falls or to minimize the impact of falls if they occur. It encompasses an individual's belief in their ability to perform activities of daily living without falling [38, 39].
Personality traits	Primarily defined according to the Five‐Factor Model [40] which includes neuroticism (emotional instability), extraversion, conscientiousness, agreeableness, and openness. To capture the broader range of evidence linking personality and falls, studies employing other validated constructs, such as the Type A behaviour pattern [41], were also included.

### 2.7 Synthesis of Results

The findings were synthesized using two complementary approaches: (a) a descriptive numerical analysis to map the distribution of studies by geographical location, study design, and outcomes examined; and (b) a narrative summary to collate key findings. The narrative synthesis focused on two aspects: (i) the relationship between personality traits and falls or fall‐related psychological concerns, and (ii) factors that mediated these relationships.

## 3 Result

### 3.1 Study Selection

**Figure 2:**
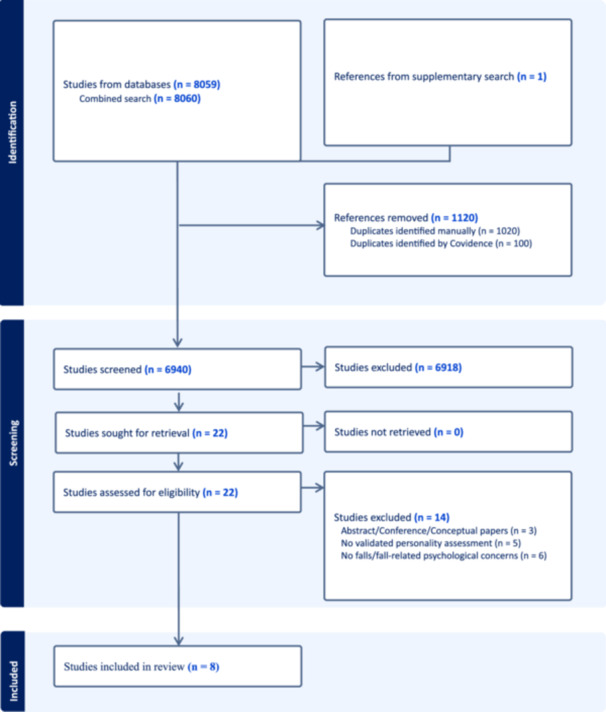
PRISMA flow diagram of study selection process.

From 8060 records identified through database and supplementary searches, eight studies met the eligibility criteria and were included in this review (Figure 2). The included studies were published between 2004 and 2024, although the search window spanned 1945–2024. Reasons for full‐text exclusions (*n* = 14) are listed in Appendix C.

### 3.2 Study Characteristics

The eight included studies comprised three longitudinal studies [19, 42, 43] and five cross‐sectional designs [20, 22, 32, 44, 45], conducted across North America, Europe, and Asia (Table 2). Collectively, they examined relationships between personality traits and falls or fall‐related psychological concerns. Sample sizes ranged from 199 to 69,846 participants, with mean ages between 67 and 79 years. All studies included adults aged ≥ 50 years, consistent with populations targeted in large‐scale cohorts such as SHARE, thereby shaping the scope of available evidence.

**Table 2:** Characteristics of the sources of evidence.

Author (year)	Country	Design	Population/setting	Personality and fall‐related psychological measures	Study aim	Main findings	Identified gaps
Mann et al. [44]	UK	Cross‐sectional	*n* = 1691; community‐dwelling female (≥ 70 years), mean age = 77.5 (SD 4.76).	EPI (neuroticism); FOF (6‐point Likert scale item).	Examine the relationship between FOF and neuroticism in older adults.	Higher neuroticism (OR 1.47 per SD increase, *p* < 0.001) and history of falls (OR 1.57, *p* < 0.001) were significant predictors of FOF.	Limited studies examining personality and FOF relationships; variability in FOF measurement.
Kloseck et al. [22]	Canada	Cross‐sectional	*n* = 199 community‐dwelling older adults (mean age 78.9, SD 7.12, 70–85 years); 76% females.	FES‐I; General questions (FOF); Personality based on descriptions by McCrae and Costa [46].	Investigate the influence of personality, confidence, health, and well‐being on engagement in daily activities among older adults.	Higher neuroticism and lower conscientiousness were associated with increased fall risk; extraversion was linked to greater community engagement.	Limited studies on personality and self‐efficacy; measurement inconsistencies.
Bower et al. [42]	US	Longitudinal prospective	*n* = 263; aged 60+ with recent hip fracture, recruited from 8 hospitals (mean age 77.54, SD 8.78 years); 75.3% female.	Mini IPIP; Short FES‐I.	Explore physical and psycho‐social risk factors of chronic FOF after hip fracture.	Higher neuroticism was associated with greater and persistent FOF post‐fracture.	Limited clinical population studies.
Canada et al. [19]	US	Longitudinal	*n* = 4,759; aged 65–99 years (mean 73.58; SD 6.5); 54.9% females (Health and Retirement Study).	MIDI	Examine personality and falls, exploring the mediation effects of health‐risk behaviours.	Neuroticism and conscientiousness predicted fall risk; mediation via physical inactivity, disease burden, smoking, and depressive symptoms.	Lack of causal data; need for longitudinal validation.
Hajek & König [47]	Europe/Israel	Cross‐sectional	*n* = 69,846 older adults (mean age 68.3, SD 9.5, 50–105 years) 57% female from SHARE.	BFI‐10	Identify personality factors increasing fall risk.	Low conscientiousness decreased fall risk (OR = 0.89); high neuroticism increased fall risk (OR = 1.16),	Need for population‐specific studies.
Turunen et al. [45]	Finland	Cross‐sectional	*n* = 314 community‐dwelling older adults (mean age 74.5, SD 3.8; 70‐85 years) 60% females.	EPI (neuroticism); FES‐I	Investigate neuroticism, FOF, and falls, including potential mediation.	Neuroticism influenced indoor falls via depressive symptoms and FOF.	Limited research explores the link between personality traits and falls, especially how psychological factors mediate this relationship.
Zhang et al. [43]	China	Longitudinal prospective	*n* = 879 community‐dwelling older adults (≥ 60 years; mean age ~67.5; 434 men, 445 women) from 3 urban communities in Nanjing.	Personality: Type A behaviour pattern (Maeda's Type A Scale, 12 items, cutoff ≥ 17 = Type A). Falls: 1‐year fall diaries + monthly follow‐up (definition: “falling to the ground or hitting an object”).	Examined whether Type A behaviour is associated with fall incidence and to compare prevalence, reasons, locations, injuries, and frequency of falls between Type A and non‐Type A older adults.	Fall incidence was higher in Type A vs. non‐Type A (27.2% vs. 19.4%); significant in men (AOR = 2.06, *p* = 0.005) but not women. Slipping/tripping is more common in Type A men; no differences in injury severity.	The Type A scale was developed for coronary heart disease, not falls; mechanisms such as risk‐taking and environmental challenges require further study.
Fan et al. [20]	China	Cross‐sectional	*n* = 407 hospitalized older adults (52.6% males; median 71; 65–77).	CBF‐PI‐B; Short FES‐I	Examine concerns about falling and personality.	Neuroticism increased FOF (β = 0.158, *p* = 0.005); extraversion reduced FOF via subjective age (β = −0.08, *p* = 0.03).	Need for diverse cultural validation.

*Note:* β, standardized regression coefficient; AOR, Adjusted Odds Ratio; BFI‐10, Big Five Inventory; CBF‐PI‐B, Chinese Big Five Personality Inventory Brief Version; EPI, Eysenck Personality Inventory; FES‐I, Falls Efficacy Scale‐International; FOF, Fear of Falling; IPIP, International Personality Item Pool; MIDI, Midlife Development Inventory; n, Total number of participants in each study; OR, Odds Ratio; *p, P*‐value; SD, Standard Deviation; SHARE, Survey of Health, Ageing and Retirement in Europe.

Among the longitudinal studies, Canada et al. [19] examined 4759 community‐dwelling older adults (mean age = 73.6) from the U.S. Health and Retirement Study to assess associations between personality traits and falls, including potential mediation by health behaviours. Bower et al. [42] followed 263 hip fracture patients (mean age = 77.5) recruited from eight hospitals to identify physical and psychosocial factors related to persistent fear of falling. Zhang et al. [43] studied 879 community‐dwelling older adults (mean age = 67.5) in China, using Maeda's Type A behaviour pattern scale to prospectively examine fall incidence over twelve months.

The five cross‐sectional studies were conducted in varied settings. Hajek and König [47] analysed data from 69,846 adults (mean age = 68.3) across several European countries and Israel using the Survey of Health, Ageing, and Retirement in Europe (SHARE) survey to evaluate Big Five personality traits in relation to falls. Mann et al. [44] surveyed 1,691 women aged ≥ 70 years in the United Kingdom to examine the link between neuroticism and fear of falling. Turunen et al. [45] investigated 314 community‐dwelling older adults in Finland, focusing on neuroticism, fear of falling, and falls, with depressive symptoms analysed as potential mediators. Fan et al. [20] studied 407 hospitalized older adults in China, exploring associations between personality traits and concerns about falling, including the role of subjective age. Finally, Kloseck et al. [22] examined 199 community‐dwelling Canadians to explore how personality, fall self‐efficacy, and perceptions of health influenced engagement in daily activities.

Across studies, neuroticism (tendency toward anxiety and worry) and conscientiousness (cautiousness and self‐discipline) were the most examined traits, while extraversion, openness, agreeableness, and Type A behaviour pattern received less consistent attention. Fear of falling and fall self‐efficacy were assessed using both standardized scales (e.g., Falls Efficacy Scale–International, Short FES‐I) and single‐item questions, reflecting heterogeneity in measurement approaches. Although a formal quality appraisal was not undertaken, most included studies clearly described their samples and used validated personality and fall‐related instruments, while longitudinal evidence remains limited.

### 3.3 Personality and Fall‐Related Assessments

Personality traits were assessed using several validated instruments (Table 3), illustrating substantial methodological variation. Instruments included the 10‐item *Big Five Inventory* (BFI‐10) [20, 47], the *Mini International Personality Item Pool* (IPIP) [42], the *Eysenck Personality Inventory* [44, 45], and the *Midlife Development Inventory* (MIDI) [19], Kloseck et al. [22] applied general descriptors from McCrae and Costa [46], whereas Zhang et al. [43] used Maeda's *Type A* b*ehaviour* pattern scale, originally designed for coronary heart disease research.

**Table 3:** Personality assessment tools used in included studies.

Study (author, year)	Instrument	Traits/constructs measured	Format/items	Notes/limitations
Canada et al. [19]	Midlife Development Inventory (MIDI)	Big Five traits	26 items, self‐report	Comprehensive measure; well‐validated, but longer format.
Bower et al. [42]	Mini International Personality Item Pool (Mini IPIP)	Neuroticism	4‐item subscale, self‐report	Brevity limits depth: only one trait captured.
Hajek & König [47]; Fan et al. [20],	Big Five Inventory–10 (BFI‐10)/Chinese BFI Brief	Big Five traits	10 items (2 per trait)	Very brief; reduced reliability, especially for openness and agreeableness.
Mann et al. [44]; Turunen et al. [45]	Eysenck Personality Inventory (EPI)	Neuroticism	Multi‐item questionnaire	Older tool; limited comparability with Big Five frameworks.
Kloseck et al. [22]	General trait questions [46]	Big Five constructs (general)	Non‐standardized, descriptive	Lower reliability; difficult to compare across studies.
Zhang et al. [43]	Type A behaviour pattern scale (Maeda, 12 items)	Type A behaviour pattern	12 items, cutoff ≥ 17 = Type A	Designed for coronary heart disease; not validated for fall risk.

Fear of falling and fall self‐efficacy were primarily assessed using the fall efficacy scale international (FES‐I) in four studies [20, 22, 42, 45]. Two studies used single‐item general questions to assess fear of falling [22, 44]. Falls were captured across all studies through self‐reported questionnaires or diaries, with recall periods ranging from 12 months [43] to multiple years [19].

As summarized in Table 3, personality instruments ranged from comprehensive inventories such as the MIDI to brief screening tools like the BFI‐10, and in one case, a health‐specific measure (Type A behaviour Scale). The variation underscores the limited standardization of personality measurement across studies and highlights the need for consistent use of validated tools in future research.

### 3.4 Relationship Among Personality, Falls, and Fall‐Related Psychological Concerns

Across studies, higher scores represented greater expression of each trait as defined by the respective instrument. For example, higher neuroticism indicated greater emotional instability, higher conscientiousness reflected stronger organization and caution, and higher extraversion reflected greater sociability and energy. Although scoring formats varied slightly, the interpretation of high and low levels was consistent across studies.

Higher neuroticism was consistently associated with an increased likelihood of falls, including indoor, outdoor, and recurrent events [19, 22, 45, 47]. Conscientiousness was inversely associated with falls, with lower scores linked to greater fall risk [19, 22]. No consistent associations were observed for extraversion, openness, or agreeableness. Zhang et al. [43] uniquely assessed the *Type A behaviour pattern*, reporting a higher fall incidence among Type A men compared with non‐Type A men (25.3% vs. 14.2%; adjusted AOR = 2.06, *p* = 0.005), with no significant association among women.

Higher neuroticism was also linked to greater fear of falling [20, 22, 42, 44, 45]. Findings for extraversion were consistent across studies, with lower extraversion associated with greater fear of falling [22] and higher extraversion linked to reduced concerns about falling [20]. Evidence connecting personality traits and fall self‐efficacy was limited to one study [22], where extraversion was positively correlated with community engagement, while agreeableness and neuroticism were linked to lower fall self‐efficacy, particularly for activities performed at home.

### 3.5 Mediating Factors in the Relationship

Several studies examined variables that may link personality traits and fall outcomes. In Canada et al. [19], the associations of conscientiousness and neuroticism with falls were partly explained by disease burden, depressive symptoms, physical inactivity, smoking status, and handgrip strength. After adjustment, conscientiousness remained significant, whereas neuroticism was no longer associated with falls. Cognitive functioning was also assessed as a potential mediator but did not account for the associations between personality and fall outcomes.

Turunen et al. [45] identified fear of falling and depressive symptoms as mediators between neuroticism and indoor/outdoor falls. Fan et al. [20] reported that subjective age mediated the association between openness, agreeableness, and fear of falling, and partially mediated the link between extraversion and fear of falling. Kloseck et al. [22]. found that perceptions of health and age were associated with both personality (particularly emotional instability and extraversion) and fall self‐efficacy, although mediation was not formally tested.

In clinical contexts, Bower et al. [42] found that hip fracture patients higher in neuroticism were more likely to report persistent fear of falling during rehabilitation. Conversely, Hajek & König [47] and Zhang et al. [43] observed direct relationships between personality and falls without analysing potential mediators. Overall, mediators assessed across studies included psychological constructs (fear of falling, depressive symptoms, subjective age) and health‐related factors (chronic disease, physical activity, smoking, handgrip strength). The variability in analytic approaches limits comparability but demonstrates growing attention to indirect associations between personality and fall‐related outcomes.

### 3.6 Findings from Studies on Specific Populations

Two studies focused on distinct older adult subgroups. Bower et al. [42]. investigated adults ≥ 65 years with hip fracture and reported that higher neuroticism was associated with greater fear of falling following rehabilitation, Mann et al. [44]. examined women aged ≥ 70 years and observed that higher neuroticism was linked to fear of falling. These subgroup findings highlight how population characteristics and health status may influence the observed associations between personality and fall‐related outcomes.

## 4 Discussion

This scoping review synthesized evidence on the relationship among personality traits, falls, and fall‐related psychological concerns in older adults. Across the eight included studies, neuroticism (emotional instability) and conscientiousness were the traits most consistently associated with fall‐related outcomes. In general, higher neuroticism was linked to greater fall risk and higher fear of falling, whereas lower conscientiousness was related to increased fall risk [19, 22, 44]. Evidence for extraversion was consistent, with lower levels linked to greater fear of falling and higher levels linked to reduced fear of falling [20, 22]. Type A behaviour was related to higher fall incidence among men only [43], while openness and agreeableness showed little consistent association. Collectively, these findings position personality as a meaningful yet under‐explored correlate of fall risk and fall‐related psychological concerns. These patterns align with broader evidence in gerontology linking personality to health behaviour, resilience, and successful aging [17, 48]. Such findings suggest that traits influencing adaptation and motivation may similarly shape fall‐related behaviours in later life.

Beyond direct associations, the included studies point to several indirect or contextual pathways through which personality traits may influence fall‐related outcomes. Health and behavioural mediators such as disease burden, depressive symptoms, physical inactivity, smoking, and handgrip strength were reported to partially explain associations between conscientiousness, neuroticism, and falls [19]. Psychological factors including fear of falling, depressive symptoms, and subjective age also emerged as potential mediators [20, 45]. Perceived health and age were linked with both personality and fall self‐efficacy, suggesting that older adults' interpretations of health and capability may shape these associations [22]. These pathways echo principles of social‐cognitive and health‐belief models, where perceived control, fall self‐efficacy, and affective regulation mediate behavioural outcomes [49, 50]. Personality traits such as conscientiousness and neuroticism may therefore influence fall‐related behaviours partly through their effects on perceived capability and motivation.

Evidence from clinical contexts similarly identified personality–psychological interactions. Among hip‐fracture patients, higher neuroticism was associated with persistent fear of falling during rehabilitation [42]. In contrast, large‐scale population studies mainly demonstrated direct associations between personality and falls without testing mediators [43, 44, 47]. Overall, current evidence indicates that personality may interact with both psychological and health‐related processes, but the diversity of analytical approaches limits comparability and precludes firm conclusions.

## 5 Implications for Practice and Research

These findings also align with current movements toward age‐friendly and person‐centred care, which emphasize tailoring interventions to “what matters” to older adults while supporting mobility, mentation, and meaningful engagement [51, 52]. Understanding personality can inform fall‐prevention strategies tailored to behavioural tendencies and emotional responses. Emotionally unstable older adults may benefit from psychological interventions (e.g., cognitive–behavioural therapy) to address anxiety and fear of falling [53]. Those lower in conscientiousness might respond better to structured education, goal‐setting, or environmental prompts encouraging safe behaviour. Wearable activity monitors and other feedback‐based technologies may enhance self‐awareness and adherence to fall‐prevention recommendations, particularly for individuals prone to avoidance or reduced confidence [54]. Socially oriented programs could also be tailored, group formats for more extraverted individuals and gradual, confidence‐building interventions for those less socially inclined.

Methodological variability was evident across studies. Personality was measured using diverse instruments, from comprehensive inventories such as the MIDI to brief versions like the BFI‐10, and in some cases with scales developed for unrelated contexts (e.g., the Type A scale). Fall‐related psychological concerns were likewise measured inconsistently. Adopting standardized, validated measures of both personality and fall‐related psychological concerns will improve comparability and meta‐analytic potential. Future work should also address current gaps: (a) the scarcity of research on fall self‐efficacy and balance confidence; (b) the under‐representation of hospitalized and post‐fracture populations; and (c) the limited number of longitudinal designs capable of clarifying temporal relationships. Incorporating broader personality models such as HEXACO [55] may further expand understanding by capturing traits beyond the traditional Big Five.

### 5.1 Limitations

Only eight studies met the inclusion criteria, despite more than 8000 records screened, reflecting a genuine evidence gap rather than restrictive eligibility parameters. The search strategy was intentionally broad, covering five major databases and an extended time frame (1945–2024) to maximize sensitivity. It also included terms such as *frailty* to capture related constructs. Many excluded papers examined associated factors (e.g., depression, anxiety) without employing a validated personality assessment, or existed only as conference abstracts. Thus, the small number of eligible studies mirrors the scarcity of research directly integrating personality and fall outcomes rather than methodological limitations.

Consistent with PRISMA‐ScR guidance, no formal critical appraisal was conducted, limiting conclusions about evidence strength [26]. Substantial heterogeneity across personality and fear of falling measures further constrained synthesis. Most studies were cross‐sectional and focused on community‐dwelling adults, limiting insight into causal relationships and clinical populations. Notably, evidence on fall self‐efficacy and balance confidence remains minimal, highlighting a key area for future inquiry.

## 6 Conclusion

This scoping review mapped current evidence on the relationship between personality traits and fall‐related outcomes among older adults. Across the eight included studies, neuroticism and conscientiousness were most consistently associated with falls and fear of falling, whereas extraversion showed a generally protective association with fear of falling, and Type A behaviour was significant only among men. Research on openness, agreeableness, fall self‐efficacy, and balance confidence remains limited. The small number of eligible studies and the heterogeneity of personality and fall‐related measures underscore the need for standardized assessment tools and expanded research across diverse populations and contexts. Overall, the findings position personality as an underexplored but potentially important determinant of fall risk and adaptation in aging, warranting further investigation through longitudinal and clinically focused studies.

## Author Contributions

**Henrietha Adandom:** conceptualization (lead), writing – original draft (lead), writing – review and editing (equal), methodology – search strategy (supporting). **Henry Nwankwo:** writing – review and editing (equal). **David Scott:** methodology – search strategy (lead), writing – review and editing (equal). **Israel Adandom:** writing – review and editing (equal). **Olayinka Akinrolie:** writing – review and editing (equal). **Adesola Odole:** supervision (supporting), writing – review and editing (equal). **Lisa Cook:** supervision (supporting), writing – review and editing (equal). **Gongbing Shan:** supervision (supporting), writing – review and editing (equal). **Oluwagbohunmi Awosoga:** supervision (lead), writing – review and editing (equal).

## Funding

The authors received no specific funding for this work.

## Conflicts of Interest

The authors declare no conflicts of interest.

## Transparency Statement

The lead author, Henrietha C. Adandom, affirms that this manuscript is an honest, accurate, and transparent account of the study being reported; that no important aspects of the study have been omitted; and that any discrepancies from the study as planned (and, if relevant, registered) have been explained.

## Supporting information


**Appendix 1:** Preferred Reporting Items for Systematic reviews and Meta‐Analyses extension for Scoping Reviews (PRISMA‐ScR) checklist [ 27]. **Appendix 2:** Details of the search strategy used in 4 databases. Appendix 3. Characteristics of excluded studies.

## Data Availability

The data for this scoping review consists of published studies retrieved from [databases searched, e.g., MEDLINE, APA PsycINFO, Web of Science, CINAHL, SPORTDiscus]. The full search strategy, including databases, search terms, and inclusion/exclusion criteria, is provided in the manuscript and supplementary materials. No new primary data were collected, and all included sources are publicly accessible.
