# Supplementary material for: Personality Traits and Their Association With Falls and Fall‐Related Psychological Concerns in Adults Aged 50 and Older: A Scoping Review
Source: Health Sci Rep. 2026 Mar 30;9(4):e72138. doi: 10.1002/hsr2.72138 (PMC13087536; doi:10.1002/hsr2.72138)
Supplement: Supplementary file 1 — Appendix 1: Preferred Reporting Items for Systematic reviews and Meta‐Analyses extension for Scoping Reviews (PRISMA‐ScR) checklist [ 27]. Appendix 2: Details of the search strategy used in 4 databases. Appendix 3. Characteristics of excluded studies. [file HSR2-9-e72138-s001.docx]

# Appendix 1. Preferred Reporting Items for Systematic reviews and Meta-Analyses extension for Scoping Reviews (PRISMA-ScR) checklist [27]

| Section | Item | PRISMA-ScR checklist item | Reported on page number |
| --- | --- | --- | --- |
| Title | 1 | Identify the report as a scoping review. | 1 |
| Abstract |  | | |
| Structured summary | 2 | Provide a structured summary that includes (as applicable) background, objectives, eligibility criteria, sources of evidence, charting methods, results, and conclusions that relate to the review questions and objectives. | 2-3 |
| Introduction |  | | |
| Rationale | 3 | Describe the rationale for the review in the context of what is already known. Explain why the review questions/objectives lend themselves to a scoping review approach. | 5 |
| Objectives | 4 | Provide an explicit statement of the questions and objectives being addressed with reference to their key elements (e.g., population or participants, concepts, and context) or other relevant key elements used to conceptualize the review questions and/or objectives. | 6 |
| Methods |  |  |  |
| Protocol and Registration | 5 | Indicate whether a review protocol exists; state if and where it can be accessed (e.g., a Web address); and if available, provide registration information, including the registration number. | 6 |
| Eligibility criteria | 6 | Specify characteristics of the sources of evidence used as eligibility criteria (e.g., years considered, language, and publication status), and provide a rationale | 6-7 |
| Information sources | 7 | Describe all information sources in the search (e.g., databases with dates of coverage and contact with authors to identify additional sources), as well as the date the most recent search was executed. | 7-8 |
| Search | 8 | Present the full electronic search strategy for at least 1 database, including any limits used, such that it could be repeated. | Appendix 2 |
| Selection of sources of evidence | 9 | State the process for selecting sources of evidence (i.e., screening and eligibility) included in the scoping review. | 8 |
| Data charting process | 10 | Describe the methods of charting data from the included sources of evidence (e.g., calibrated forms or forms that have been tested by the team before their use, and whether data charting was done independently or in duplicate) and any processes for obtaining and confirming data from investigators. | 8-9 |
| Data items | 11 | List and define all variables for which data were sought and any assumptions and simplifications made. | Table 1 |
| Critical appraisal of individual sources of evidence | 12 | If done, provide a rationale for conducting a critical appraisal of included sources of evidence; describe the methods used and how this information was used in any data synthesis (if appropriate). | N/A |
| Synthesis of results | 13 | Describe the methods of handling and summarizing the data that were charted. | 9 |
| Results |  | | |
| Selection of sources of evidence | 14 | Give numbers of sources of evidence screened, assessed for eligibility, and included in the review, with reasons for exclusions at each stage, ideally using a flow diagram. | 10 |
| Characteristics of sources of evidence | 15 | For each source of evidence, present characteristics for which data were charted and provide the citations. | Table 2 |
| Critical appraisal within sources of evidence | 16 | If done, present data on critical appraisal of included sources of evidence (see item 12). | N/A |
| Results of individual sources of evidence | 17 | For each included source of evidence, present the relevant data that were charted that relate to the review questions and objectives. | Table 2 |
| Synthesis of results | 18 | Summarize and/or present the charting results as they relate | 10-12 |
| Discussion |  | | |
| Summary of evidence | 19 | Summarize the main results (including an overview of concepts, themes, and types of evidence available), link to the review questions and objectives, and consider the relevance to key groups. | 13-14 |
| Limitations | 20 | Discuss the limitations of the scoping review process. | 14 |
| Conclusions | 21 | Provide a general interpretation of the results with respect to the review questions and objectives, as well as potential implications and/or next steps. | 14-15 |
| Funding |  | | |
| Funding | 22 | Describe sources of funding for the included sources of evidence, as well as sources of funding for the scoping review. Describe the role of the funders of the scoping review. | 16 |

# Appendix 2. Details of the search strategy used in 4 databases

**Web of Science Search Strategy**

1. TS=(emotion* NEAR/5 stab*)
2. TS=(personalit* or extraver* or extrover* or introver* or agreeab* or conscientious* or neuroti* or openness)
3. TS=(“big five” or “five factor”)
4. #3 OR #2 OR #1
5. TS=(falls or faller* or frail*)
6. TS=((fear* or fright* or afraid or concern* or efficacy) NEAR/5 fall*)
7. TS=(ptophob*)
8. TS=(balanc* NEAR/5 confiden*)
9. #8 OR #7 OR #6 OR #5
10. #4 AND #9
11. #4 AND #9 and English (Languages)

**APA PsycINFO Search Strategy**

1. exp Personality/
2. (personalit* or extraver* or extrover* or introver* or agreeab* or conscientious* or neuroti* or openness).tw.
3. (emotion* adj5 stab*).tw.
4. (“big five” or “five factor”).tw.
5. or/1-4
6. falls/
7. exp health impairments/
8. equilibrium/
9. (falls or faller* or frail*).tw.
10. ((fear* or fright* or afraid or concern* or efficacy) adj5 fall*).tw.
11. ptophob*.tw.
12. (balanc* adj5 confiden*).tw.
13. or/6-12
14. 5 and 13
15. limit 14 to english language

**CINAHL Search Strategy**

1. (MH "Personality+")
2. personalit* or extraver* or extrover* or introver* or agreeab* or conscientious* or neuroti* or openness
3. emotion* N5 stab*
4. "big five" or "five factor"
5. S1 OR S2 OR S3 OR S4
6. (MH "Accidental Falls")
7. (MH "Frailty Syndrome")
8. (MH "Frail Elderly")
9. falls or faller* or frail*
10. (fear* or fright* or afraid or concern* or efficacy) N5 fall*
11. ptophob*
12. balanc* N5 confiden*
13. S6 OR S7 OR S8 OR S9 OR S10 OR S11 OR S12
14. S5 AND S13
15. S5 AND S13 [with English language filter applied]

**SPORTDiscus Search Strategy**

1. DE "PERSONALITY" OR DE "AGGRESSION (Psychology)" OR DE "BODY image" OR DE "CHARACTER" OR DE "CONSCIENTIOUSNESS" OR DE "IDENTITY (Psychology)" OR DE "MOOD (Psychology)" OR DE "PSYCHOLOGICAL resilience" OR DE "SOCIABILITY"
2. personalit* or extraver* or extrover* or introver* or agreeab* or conscientious* or neuroti* or openness
3. emotion* N5 stab*
4. S1 OR S2 OR S3
5. DE "ACCIDENTAL falls"
6. falls or faller* or frail*
7. (fear* or fright* or afraid or concern* or efficacy) N5 fall*
8. ptophob*
9. balanc* N5 confiden*
10. S5 OR S6 OR S7 OR S8 OR S9
11. S4 AND S10
12. S4 AND S10 [with English language filter applied]

Note. MeSH = medical subject heading; exp = used with a MeSH term to include all narrower MeSH terms; .tw. = field codes for text word; adj# = search for records with terms within # words of each other; quotation marks (e.g., “five factor”) indicate a phrase search; * after keyword indicates truncation (e.g., fall* will retrieve “fall”, “falls”, “falling”, etc.

# Appendix 3. Characteristics of excluded studies

| Author (Year) | Title | Reason for Exclusion |
| --- | --- | --- |
| Agmon & Armon, (2016) | A cross-sectional study of the association between mobility test performance and personality among older adults. | Examined personality ↔ mobility (TUG); no falls or FRPC outcomes. |
| Agmon et al.(2018) | The role of gender in the association between personality and task priority in older adults' dual tasking while walking. | Focused on dual task walking and task priority; no falls or FRPC outcomes. |
| Bosma et al.(2004) | Demographic, health-related and psychosocial predictors of changes in depressive symptoms and anxiety in late middle-aged and older persons with fall-related injuries. | Outcome = depression/anxiety trajectories; did not analyze personality ↔ falls/ FRPC. |
| Canada et al.(2021) | Cross-sectional and prospective association between personality traits and IADL/ADL limitations. | Outcome = functional limitations (ADL/IADL); no falls/FRPC |
| Elfering et al.(2013) | Busy at work and absent-minded at home: Mental workload, cognitive failure, and domestic falls. | no personality ↔ falls/ FRPC focus |
| Faulkner et al.(2009) | Does conscientiousness protect against recurring falls in older men? | Conference abstract only (no peer-reviewed full text) |
| Kloseck et al.(2008) | Can personality theory help us understand risk of falls? | Conceptual/theoretical article; no primary data |
| LeMonda et al.(2015) | The association between high neuroticism-low extraversion and dual-task performance during walking while talking in non-demented older adults | Outcome = dual-task gait/cognition; no falls/ FRPC outcomes. |
| Ndubuaku et al.(2023) | Do personality traits predict mobility outcomes among community-dwelling older adults in Nigeria? | Examined personality ↔ mobility; no falls or FRPC outcomes |
| Ni Mhaolain et al.(2012) | Depression: A modifiable factor in fearful older fallers transitioning to frailty? | Focused on depression among fearful fallers; no analysis of personality traits. |
| Sun et al.(2022) | Longitudinal association between personality traits and homebound status in older adults: results from the National Health and Aging Trends Study | Outcome = homebound status; no falls/ FRPC. |
| Trajanoska et al.(2020) | Genetic basis of falling risk susceptibility in the UK Biobank Study. | GWAS on falls; no validated psychological personality measure. |
| Vetter & Ford (1989) | Anxiety and depression scores in elderly fallers. | Outcome = anxiety/depression; no validated personality assessment |
| Yozawitz & Holtzer (2016) | The association of extraversion and neuroticism with prevalence of fear of falling in the elderly | Conference abstract only (no peer-reviewed full text) |

Note. FRPC = fall-related psychological concerns; TUG = time up and go; ADL = activities of daily living; IADL = instrumental activities of daily living; GWAS = genome-wide association studies.

Reference of excluded studies

1. M. Agmon, G. Armon, “A cross-sectional study of the association between mobility test performance and personality among older adults,” *BMC Geriatr*ic 16, no. 1 (2016):105.
2. M. Agmon, G. Armon, S. Denesh. et al., “The role of gender in the association between personality and task priority in older adults’ dual-tasking while walking,” BMC Geriatrics 18, no. 1 (2018):1
3. H. Bosma, R. Sanderman, W. Scaf-Klomp. et al., “Demographic, health-related and psychosocial predictors of changes in depressive symptoms and anxiety in late middle-aged and older persons with fall-related injuries,” Psychology & Health 19, no. 1 (2004): 103–115.
4. B. Canada, Y. Stephan, H. Fundenberger. et al., “Cross-sectional and prospective association between personality traits and IADL/ADL limitations,” Psychology and Aging 36, no. 3 (2021): 309–321.
5. A. Elfering, S. Grebner, C. Boillat, “Busy at work and absent-minded at home: mental workload, cognitive failure, and domestic falls,” Swiss Journal of Psychology 72, no. 4 (2013): 219–228.
6. K. Faulkner, S. Studenski, M. Scheier. et al., “Does Conscientiousness protect against recurring falls in polder men,” *Gerontologist* 49 (2009): 244.
7. M. Kloseck, R. G. Crilly, and M. Gibson, “Can personality theory help us understand risk of falls?,” *Journal of Rehabilitation Research and Development* 45, no. 8 (2008):1125.
8. B. C. LeMonda, J. R. Mahoney, J. Verghese. et al., “The association between high neuroticism–low extraversion and dual-task performance during walking while talking in non-demented older adults,” Journal of the International Neuropsychological Society 21, no. 7 (2015): 519–530.
9. M. Ndubuaku, C. Ikele, E. Nwachukwu. et al., “Do personality traits predict mobility outcomes among community-dwelling older adults in Nigeria?,” *Physical & Occupational Therapy in Geriatrics* 41, no. 3 (2023): 466–482.
10. A. M. Ní Mhaoláin, C. W. Fan, R. Romero-Ortuno. et al., “Depression: a modifiable factor in fearful older fallers transitioning to frailty?,” *International Journal of Geriatric Psychiatry* 27, no. 7 (2012): 727–733.
11. X. Sun, S. Tang, C. E. Miyawaki. et al., “Longitudinal association between personality traits and homebound status in older adults: results from the National Health and Aging Trends Study,” *BMC Geriatrics* 22 (2022): 93.
12. K. Trajanoska, L. J. Seppala, C. Medina-Gomez. et al., “Genetic basis of falling risk susceptibility in the UK Biobank study,” *Communications Biology* 3 (2020): 543.
13. N. J. Vetter, D. Ford, “Anxiety and depression scores in elderly fallers,” *International Journal of Geriatric Psychiatry* 4, no. 3 (1989): 159–163.
14. L. R. Yozawitz, R. Holtzer, “The association of extraversion and neuroticism with prevalence of fear of falling in the elderly,” *Gerontologist* 56 (2016).
